# Supplementary material for: Association of Breastfeeding for the First Six Months of Life and Autism Spectrum Disorders: A National Multi-Center Study in China
Source: Nutrients. 2021 Dec 23;14(1):45. doi: 10.3390/nu14010045 (PMC8746572; doi:10.3390/nu14010045)
Supplement: Supplementary file 1 [file nutrients-14-00045-s001.zip › nutrients-1518372-supplementary.pdf]

SUPPLEMENT

Table S1 Overview of the studies about the association between breastfeeding and risk of ASD

Table S2 Comparison of demographic characteristics of participants with or without the information of questionnaires

Table S3 Demographic information and maternal risk factors during pregnancy between toddlers with and without ASD

Table S4 Comparison of breastfeeding status under the first six months among toddlers with and without ASD

Table S5 Prevalence of ASD associated with breastfeeding during the first six months among toddlers

Table S6 The association between breastfeeding status under the first six months by using Firth's Bias-Reduced Logistic Regression

Table S7 The associations between breastfeeding status under the first six months and ASD when restricting toddlers in full-term birth

Table S8 The associations between breastfeeding status under the first six months and ASD when restricting mothers of toddlers without complications during pregnancy

Table S9 The associations between breastfeeding status under the first six months and ASD when restricting mothers of toddlers without overweight or obesity before pregnancy.

Table S1. Overview of the studies about the association between breastfeeding and risk of ASD.

Year	Author	Country	group	Sample size	Age	Outcome definition		Exposure definition	OR/ RR (95%CI)
						Diagnostic procedure	Diagnostic criterion		
**Case-control**									
2018	Bittker[1]	USA	ASD<br>Controls	1,001<br>514	3-12 years	Parent-report	NA	Breastfeeding (months)	0.948 (0.932-0.965)
2018	Manohar[2]	India	ASD<br>TD siblings	30<br>30	2-6 years	Standard assessment (CARS)	DSM-5	Compares exclusive breastfeeding up to 6 months with no exclusive breastfeeding up to 6 months	0.167 (0.025–0.65)
2016	Say[3]	Turkey	ASD<br>ADHD<br>Controls	98<br>96<br>79	3-18 years	Diagnostic procedure by an expert child and adolescent psychiatrist	DSM-Ⅳ	Compares never breastfeeding with 1 month, 3 months, 6 months, 12 months and ≥ 24 months	0.23 (0.02–2.06), 0.22 (0.02–1.92), 0.2 (0.02–1.76), 0.21 (0.02–1.98) and 0.53 (0.04–6.65)
2014	Field[4]	USA	ASD<br>ADHD<br>Controls	31<br>81<br>612	NA	Diagnostic procedure by an expert child and adolescent psychiatrist	DSM-Ⅳ-TR	Compares benefits of breastfeeding < 4 months and ≥ 4 months	Unreported
2014	George[5]	India	ASD<br>Controls	143<br>200	2-6 years	Standard assessment (CARS)	NA	Compares breastfeeding duration nil / < 6 month with ≥ 6 months	3.4 (1.28-8.99)
2014	Brown[6]	Australia	ASD<br>TD siblings	19<br>23	NA	Administrative databases	NA	Compared first hour feeding with late initiation of breastfeeding	0.26(0.07-0.89)
2014	Shafai[7]	USA	PDD<br>Contorls	60<br>85	ND	Parent-report	NA	Assess benefits of breastfeeding < 2 months, 2–4 months, 4–6 months, 6–9	Unreported

								months, 9–12 months, 12–15 months, 15–18 months, 18–24 months, and ≥ 24 months	
2012	Al-Farsi[8]	Oman	ASD<br>Controls	102<br>102	3-14 years	Standard assessment (CARS)	DSM-IV-TR	Compare late initiation of breast-feeding with early initiation of breastfeeding<br>Compared non-intake of colostrum with intake of colostrum<br>Compared duration of exclusive breastfeeding with never breastfeeding<br>Compared continued breastfeeding with non-continued breastfeeding	1.48(1.01-3.1)<br>1.7(1.03-4.3)<br>*P* for trend = 0.04<br>P for trend = 0.001
2012	Bawono[9]	Indonesia	ASD<br>Controls	52<br>104	3-10 years	Diagnostic procedure by an expert child and adolescent psychiatrist	DSM-IV-TR	Compares ≥ 6 months breastfeeding with never breastfeeding<br>Compares never exclusive breastfeeding with breastfeeding 0–6 months, 6-<12 months and ≥ 12 months	2.01 (1.02-3.96)<br>5.75 (1.4-23.49)<br>2.17 (0.73-6.46)<br>1.59 (0.54-4.62)
2006	Schultz[10]	USA	ASD<br>Controls	861<br>123	7.8±3.9 years<br>7.4±4.5 years	Parent-report	NA	Compares never breastfeeding with < 2 months, 2–6 months, and ≥ 6 months	0.69 (0.32–1.48), 0.51 (0.24–1.1), and 0.4 (0.230.71)
1989	Tanoue[11]	Japan	Infantile autism<br>Controls	145<br>224	3 years	Diagnostic procedure by an expert	DSM-III	Compares never breastfeeding with < 1 week, 1 week - 1	0.43 (0.16–1.15), 0.24 (0.11-0.52),

						child and adolescent psychiatrist		month, 1–2 months, 2–3 months, 3–6 months, 6–12 months, and ≥ 12 months	0.23 (0.10-0.51), and 0.27 (0.12-0.57)
	1988	Burd[12]	USA	PDD<br>Controls	50<br>50	9.0±4.6 years<br>8.8±4.7 years	Diagnostic procedure by an expert child and adolescent psychiatrist	DSM-III	NA	Unreported
**Cohort**									
2018	Lemcke[13]	Denmark.	ASD<br>ID<br>Total	973<br>300<br>76,322	Men age = 11 years	NA	ICD-10	Give up exclusive breastfeeding within the first 3 months<br>Continued breast-feeding after 6 months	HR=1.8-1.9<br>HR=0.7-0.8
2011	Dodds[14]	Canada	ASD<br>Normal	924<br>128,809	1-17 years	Administrative databases	ICD-9 or ICD 10	Compares ever breastfeeding with never breastfeeding at discharge	1.15 (1.01-1.31)
**Cross-sectional**									
2021	Kim[15]	Korea	ASD<br>Total	606<br>374,074	6 months till 10 years of age	Administrative databases	ICD-10	Compared exclusive breastfeeding with formular feeding during the first 4 to 6 months	0.72 (0.57-0.89)
2021	Jing[16]	China	autistic trait<br>Total	2074<br>67,578	4.61±0.88 years	Screening by Autism Behavior Checklist (ABC)	Undiagnosed	Compared exclusive breastfeeding or mixed feeding with never breastfeeding	0.773 (0.676–0.884)
2019	Soke[17]	USA	ASD<br>Controls	673<br>876	30-68 months	Screening and diagnostic procedure with standard assessment	DSM-Ⅳ	Compared breastfeeding initiation with never breastfeeding<br>Compared duration of breastfeeding in the high tertile(12 months) with	0.88 (0.60-1.28)<br>0.61(0.45-0.84)<br>0.72 (0.54-0.98)

						(ADOS, ADI-R)		low(<6 months) or the middle(6-<12 months)	
2017	Boucher[18]	Spanish	Autistic trait	1,346	4-6.9 year	Screening by the Spanish version of the Childhood Autism Spectrum Test (CAST)	Undiagnosed	Compared duration of any breastfeeding(months)<br>Compared duration of predominant breastfeeding(months)<br>Compared duration of exclusive breastfeeding (months)	1.00(0.95,1.05)<br>0.98 (0.87,1.10)<br>1.02 (0.86-1.22)
2016	Ravi[19]	India	High-risk ASD<br>Low/Medium ASD	33<br>317	16-30 months	Screening by Modified Checklist of Autism in Toddlers, Revised(M-CHAT-R)	Undiagnosed	Compared late initiation of breastfeeding and early initiation<br>Compared exclusive breastfeeding in the first 6 months with no	3.9 (1.83-8.39)<br>0.2 (0.05-0.91)
2015	Husk[20]	USA	ASD<br>Controls	391<br>37,901	2-5years	Parent-report	DSM-IV	Any partial breastfeeding<br>Exclusive breastfeeding for 6 months<br>Each additional month of partial breastfeeding<br>Each additional month of exclusive breastfeeding	0.68 (0.4-1.3)<br>0.74 (0.3-1.7)<br>1.03 (0.97-1.10)<br>1.04(.096-1.13)
2011	Shamberger[21]	USA	ASD<br>Controls	NA	3–21 years	NA	NA	Compares benefits of ever breastfeeding, at 3 months, 6 months, and 12 months	Unreported

Abbreviations: ASD, autism spectrum disorder; DSM- 5, Diagnostic and Statistical Manual of Mental Disorders, Fifth Edition; DSM-Ⅳ-TR, the text revision of the fourth edition of the Diagnostic and Statistical Manual of Mental Disorders; DSM-Ⅳ, Diagnostic and Statistical Manual of Mental Disorders, Fourth Edition; DSM-III, Diagnostic and Statistical Manual of Mental Disorders, Third Edition; ICD-9, International Classification of

Diseases, Ninth Revision; ICD-10, International Classification of Diseases, 10th Revision;

Table S2 Comparison of demographic characteristics of participants with or without the information of questionnaires

Characteristics*	Children without questionnaires (N=1,879)	Children with questionnaires (N =6,049)	Overall (N =7,928)	*P* value
	No. (%)/Mean (SD)	No. (%)/Mean (SD)	No. (%)/Mean (SD)	
**Child age, months**	22.7 (3.8)	22.7 (4.1)	22.7 (4.0)	.71
**Child gender**				
Male	1,027 (55.7%)	3,364 (55.6%)	4,391 (55.6%)	.97
Female	818 (44.3%)	2,685 (44.4%)	3,503 (44.4%)	
**Only child**				
Yes	514 (29.1%)	1,772 (29.3%)	2,286 (29.2%)	.87
No	1,253 (70.9%)	4,277 (70.7%)	5,530 (70.8%)	
**Maternal education***				.07
Less than high school	441 (24.1%)	1,045 (17.3%)	1,486 (18.9%)	
Completed high school	962 (52.5%)	3,188 (52.7%)	4,150 (52.7%)	
College degree	315 (17.2%)	1,407 (23.3%)	1,722 (21.8%)	
Advanced degree	115 (6.3%)	409 (6.8%)	524 (6.6%)	

Abbreviations: SD, Standard deviation

*Among those who did not have a questionnaire, 34 were missing in gender, 112 were missing in only child and 148 were missing in mother's education level. The information was complete among those who had the questionnaire.

Table S3 Demographic information and maternal risk factors during pregnancy between toddlers with and without ASD

Characteristic	Mean (SD) /No. (%)			*P* value
	Children without ASD (N=5,978)	Children with ASD (N=71)	Total sample (N=6,049)	
**Child age (month)**	22.7±4.1	24.3±3.6	22.7±4.1	**< .01**
**Child gender**				
Male	3,302(55.2)	62(87.3)	3,364(55.6)	**< .01**
Female	2,676(44.8)	9(12.7)	2,685(44.4)	
**Only child**				
Yes	4,229(70.7)	48(67.6)	4,277(70.7)	.56
No	1,749(29.3)	23(32.4)	1,772(29.3)	
**Preterm birth**				
Yes	503(8.4)	4(5.6)	507(8.4)	.40
No	5,475(91.6)	67(94.4)	5,542(91.6)	
**Maternal age**				
≤24 years	1,031 (17.2)	14 (19.7)	1,045 (17.3)	.94
25-29 years	3,151 (52.7)	37 (52.1)	3,188 (52.7)	
30-34 years	1,391 (23.3)	16 (22.5)	1,407 (23.3)	
≥35 years	405 (6.8)	4 (5.6)	409 (6.8)	
**Maternal education level**				
Primary school and below	661 (11.1)	16 (22.5)	677 (11.2)	**< .01**
Middle school	1,269 (21.2)	20 (28.2)	1,289 (21.3)	
College degree	3,737 (62.5)	31 (43.7)	3,768 (62.3)	
Advanced degree	311 (5.2)	4 (5.6)	315 (5.2)	
**Yearly household income**				
≤¥100, 000	2,122 (35.5)	31 (43.7)	2,153 (35.6)	.35
¥100, 000-¥300, 000	2,293 (38.4)	21 (29.6)	2,314 (38.3)	
≥¥300, 000	457 (7.6)	7 (9.9)	464 (7.7)	
Not reported	1,106 (18.5)	12 (16.9)	1,118 (18.5)	
**Ethnic background**				
Han	5,605 (93.8)	69 (97.2)	5,674 (93.8)	.23
Other	373 (6.2)	2 (2.8)	375 (6.2)	
**Complications during pregnancy**				
Yes	849 (14.2)	11 (15.5)	860 (14.2)	.76
No	5,129 (85.8)	60 (84.5)	5,189 (85.8)	
**Depression during pregnancy**				
Yes	1459 (24.4)	27 (38.0)	1,486 (24.6)	**< .01**
No	4,519 (75.6)	44 (62.0)	4,563 (75.4)	
**Second-hand smoke exposure during pregnancy**				
Yes	825 (13.8)	18 (25.4)	843 (13.9)	**<.01**
No	5,153 (86.2)	53 (74.6)	5,206 (86.1)	
**Pre-pregnancy overweight/obesity**				
Yes	465 (7.8)	7 (9.9)	472 (7.8)	.52
No	5,513 (92.2)	64 (90.1)	5,577 (92.2)	

Abbreviations: SD, Standard deviation

Table S4 Comparison of breastfeeding status under the first six months among toddlers with and without ASD

	Total sample (N=6049) N (%)	Toddlers without ASD (N=5,978) N (%)	Toddlers with ASD (N=71) N (%)	*P* value
Exclusive breastfeeding	2,950 (48.8)	2,929 (49.0)	21(29.6)	<.01
Partial breastfeeding	2,551 (42.2)	2,514 (42.1)	37(52.1)	
Not breastfeeding	548 (9.1)	535 (8.9)	13(18.3)	

Table S5 Prevalence of ASD associated with breastfeeding during the first six months among toddlers

	No.	Toddlers with ASD
		n (%)
Total	6,049	71(1.2)
Breastfeeding status for the first six months		
Exclusive breastfeeding	2,950	21(0.7)
Partial breastfeeding	2,551	37(1.5)
Not breastfeeding	548	13(2.4)

Table S6 The associations between breastfeeding status under the first six months by using Firth's Bias-Reduced Logistic Regression

	Adjusted model	
	OR (95%CI)	*P* value
Breastfeeding status for the first six months		
Exclusive breastfeeding	1[Reference]	
Partial breastfeeding	1.53(0.90, 2.68)	0.12
Not breastfeeding	**2.35(1.12, 4, 75)**	**0.02**

The model was adjusted for child's age, gender, only child, maternal age, maternal education level, yearly household income, ethnic background, study area, preterm birth, pregnancy information (complications and depression during pregnancy), second-hand smoke exposure and overweight/obesity before pregnancy.

Table S7 The associations between breastfeeding status under the first six months and ASD when restricting toddlers in full-term birth

	Adjusted model	
	OR (95%CI)	*P* value
Breastfeeding status for the first six months		
Exclusive breastfeeding	1[Reference]	
Partial breastfeeding	1.52(0.87, 2.73)	0.15
Not breastfeeding	**2.77(1.30, 5.73)**	**0.01**

The model was adjusted for child's age, gender, only child, maternal age, maternal education level, yearly household income, ethnic background, study area, pregnancy information (complications and depression during pregnancy), second-hand smoke exposure and overweight/obesity before pregnancy.

Table S8 The associations between breastfeeding status under the first six months and ASD when restricting mothers of toddlers without complications during pregnancy

	Adjusted model	
	OR (95%CI)	*P* value
Breastfeeding status for the first six months		
Exclusive breastfeeding	1[Reference]	
Partial breastfeeding	1.66(0.92, 3.11)	0.10
Not breastfeeding	**2.61(1.18, 5.64)**	**0.02**

The model was adjusted for child's age, gender, only child, maternal age, maternal education level, yearly household income, ethnic background, study area, preterm birth, second-hand smoke exposure and overweight/obesity before pregnancy.

Table S9 The associations between breastfeeding status under the first six months and ASD when restricting mothers of toddlers without overweight or obesity before pregnancy.

	Adjusted model	
	OR (95%CI)	*P* value
Breastfeeding status for the first six months		
Exclusive breastfeeding	1[Reference]	
Partial breastfeeding	1.64(0.92, 3.03)	0.10
Not breastfeeding	**2.67(1.23, 5.68)**	**0.01**

The model was adjusted for child's age, gender, only child, maternal age, maternal education level, yearly household income, ethnic background, study area, preterm birth, pregnancy information (complications and depression during pregnancy), second-hand smoke exposure.

Reference

1. Bittker, S.S.; Bell, K.R. Acetaminophen, antibiotics, ear infection, breastfeeding, vitamin D drops, and autism: an epidemiological study. *Neuropsychiatr Dis Treat* **2018**, *14*, 1399-1414, doi:10.2147/NDT.S158811.
2. Manohar, H., Pravallika, M., Kandasamy, P., Chandrasekaran, V., & Rajkumar, R. P. . Role of Exclusive Breastfeeding in Conferring Protection in Children At-Risk for Autism Spectrum Disorder: Results from a Sibling Case-control Study. *Journal of neurosciences in rural practice* **2018**, *9*, 132-136, doi: https://doi.org/10.4103/jnrp.jnrp_331_17.
3. Say, G.N.; Karabekiroglu, K.; Babadagi, Z.; Yuce, M. Maternal stress and perinatal features in autism and attention deficit/hyperactivity disorder. *Pediatr Int* **2016**, *58*, 265-269, doi:10.1111/ped.12822.
4. Field, S.S. Interaction of genes and nutritional factors in the etiology of autism and attention deficit/hyperactivity disorders: a case control study. *Med Hypotheses* **2014**, *82*, 654-661, doi:10.1016/j.mehy.2014.02.021.
5. George, B.; Padmam, M.S.; Nair, M.K.; Leena, M.L.; Russell, P.S. CDC Kerala 14: Early child care practices at home among children (2-6 y) with autism--a case control study. *Indian J Pediatr* **2014**, *81 Suppl 2*, S138-141, doi:10.1007/s12098-014-1602-5.
6. Brown, C.M.; Austin, D.W.; Busija, L. Observable essential fatty acid deficiency markers and autism spectrum disorder. *Breastfeed Rev* **2014**, *22*, 21-26.
7. Shafai, T.; Mustafa, M.; Hild, T.; Mulari, J.; Curtis, A. The association of early weaning and formula feeding with autism spectrum disorders. *Breastfeed Med* **2014**, *9*, 275-276, doi:10.1089/bfm.2013.0104.
8. Al-Farsi, Y.M.; Al-Sharbati, M.M.; Waly, M.I.; Al-Farsi, O.A.; Al-Shafaee, M.A.; Al-Khaduri, M.M.; Trivedi, M.S.; Deth, R.C. Effect of suboptimal breast-feeding on occurrence of autism: a case-control study. *Nutrition* **2012**, *28*, e27-32, doi:10.1016/j.nut.2012.01.007.
9. Bawono, K.D.H., E.S.; Wandita S. Breastfeeding as a protective factor against autism. *JURNAL GIZI KLINIK INDONESIA* **2012**, *8*, 166-171.
10. Schultz, S.T.; Klonoff-Cohen, H.S.; Wingard, D.L.; Akshoomoff, N.A.; Macera, C.A.; Ji, M.; Bacher, C. Breastfeeding, infant formula supplementation, and Autistic Disorder: the results of a parent survey. *Int Breastfeed J* **2006**, *1*, 16, doi:10.1186/1746-4358-1-16.
11. Tanoue, Y.; Oda, S. Weaning time of children with infantile autism. *J Autism Dev Disord* **1989**, *19*, 425-434, doi:10.1007/BF02212940.
12. Burd, L.; Fisher, W.; Kerbeshian, J.; Vesely, B.; Durgin, B.; Reep, P. A comparison of breastfeeding rates among children with pervasive developmental disorder, and controls. *J Dev Behav Pediatr* **1988**, *9*, 247-251.
13. Lemcke, S.; Parner, E.T.; Bjerrum, M.; Thomsen, P.H.; Lauritsen, M.B. Early Regulation in Children Who Are Later Diagnosed with Autism Spectrum Disorder. A Longitudinal Study within the Danish National Birth Cohort. *Infant Ment Health J* **2018**, *39*, 170-182, doi:10.1002/imhj.21701.
14. Dodds, L.; Fell, D.B.; Shea, S.; Armson, B.A.; Allen, A.C.; Bryson, S. The role of prenatal, obstetric and neonatal factors in the development of autism. *J Autism Dev Disord* **2011**, *41*, 891-902, doi:10.1007/s10803-010-1114-8.
15. Kim, J.H.; Lee, S.W.; Lee, J.E.; Ha, E.K.; Han, M.Y.; Lee, E. Breastmilk Feeding during the First 4 to 6 Months of Age and Childhood Disease Burden until 10 Years of Age. *Nutrients* **2021**, *13*, doi:10.3390/nu13082825.
16. Chen, J.; Strodl, E.; Huang, L.H.; Chen, J.Y.; Liu, X.C.; Yang, J.H.; Chen, W.Q. Associations between Prenatal Education, Breastfeeding and Autistic-Like Behaviors in Pre-Schoolers. *Children (Basel)* **2021**, *8*, doi:10.3390/children8020124.
17. Soke, G.N.; Maenner, M.; Windham, G.; Moody, E.; Kaczaniuk, J.; DiGuiseppi, C.; Schieve, L.A. Association Between Breastfeeding Initiation and Duration and Autism Spectrum Disorder in Preschool Children Enrolled in the Study to Explore Early Development. *Autism Res* **2019**, *12*, 816-829, doi:10.1002/aur.2091.
18. Boucher, O.; Julvez, J.; Guxens, M.; Arranz, E.; Ibarluzea, J.; Sanchez de Miguel, M.; Fernandez-Somoano, A.; Tardon, A.; Rebagliato, M.; Garcia-Esteban, R.; et al. Association between breastfeeding duration and cognitive development, autistic traits and ADHD symptoms: a multicenter study in Spain. *Pediatr Res* **2017**, *81*, 434-442, doi:10.1038/pr.2016.238.
19. Ravi, S.; Chandrasekaran, V.; Kattimani, S.; Subramanian, M. Maternal and birth risk factors for children screening positive for autism spectrum disorders on M-CHAT-R. *Asian J Psychiatr* **2016**, *22*, 17-21, doi:10.1016/j.ajp.2016.04.001.
20. Husk, J.S.; Keim, S.A. Breastfeeding and Autism Spectrum Disorder in the National Survey of Children's Health.

Epidemiology **2015**, *26*, 451-457, doi:10.1097/EDE.0000000000000290.

21. Shamberger, R.J. Autism rates associated with nutrition and the WIC program. *J Am Coll Nutr* **2011**, *30*, 348-353, doi:10.1080/07315724.2011.10719978.
